# An authentic learner-centered planetary health assignment: A five-year evaluation of student choices to address Sustainable Development Goal 13 (*Climate Action*)

**DOI:** 10.3389/fpubh.2022.1049932

**Published:** 2022-11-03

**Authors:** Michelle McLean, Charlotte Phelps, Jessica Smith, Neelam Maheshwari, Vineesha Veer, Dayna Bushell, Richard Matthews, Belinda Craig, Christian Moro

**Affiliations:** Faculty of Health Sciences and Medicine, Bond University, Gold Coast, QLD, Australia

**Keywords:** Sustainable Development Goals, climate change, global citizenship, planetary health, sustainable healthcare education, medicine, planetary citizenship, health science

## Abstract

A Code Red has been declared for the planet and human health. Climate change (e.g., increasing temperatures, adverse weather events, rising sea levels) threatens the planet's already declining ecosystems. Without urgent action, all of Earth's inhabitants face an existential threat. Health professions education should therefore prepare learners to not only practice in a changing world, but authentic educational activities should also develop competencies for global and planetary citizenship. Planetary health has been integrated across the five-year Bond University (Australia) medical curriculum. It begins in the second week of Year 1 and ends with a session on Environmentally Sustainable Healthcare in the General Practice rotation in the final year. The purpose of this article is to describe the outcomes of the first 5 years (2018–2022) of a learner-centered planetary health assignment, underpinned by the 2030 United Nations (UN) Sustainable Development Goals (SDGs), in the second year of a five-year medical program. Using systems and/or design thinking with a focus on SDG13 (*Climate Action*) plus a second SDG of choice, self-selected teams of 4–6 students submit a protocol (with feedback) to develop a deliverable “product” for an intended audience. Data analysis of the first 5 years of implementation found that the most frequently selected SDGs in addition to SDG13 were: SDG12 *Sustainable Production and Consumption* (41% of teams), mostly relating to healthcare emissions and waste; SDG3 *Health and Well-being* (22%), generally involving the impact of air pollution; and SDG6 *Clean Water and Sanitation* (15%). A survey at the concluding conference garnered student feedback across various criteria. The planetary health assignment is authentic in that teams provide solutions to address climate change. Where appropriate, final “products” are sent to local or federal ministers for consideration (e.g., policy proposals) or integrated into the curriculum (e.g., learning modules). We believe that the competencies, attitudes, and values fostered through engagement with planetary health. Throughout the medical program, as evidenced by their evaluations, stands students in good stead to be change agents, not only in clinical practice but in society. An awareness has been created about the need for planetary citizenship in addition to global citizenship.

## Introduction

### Background and rationale for the educational activity innovation

A Code Red has been declared for the planet and human health (1–4). Climate change (e.g., increasing temperatures, adverse weather events, rising sea levels) threatens the planet's already declining ecosystems. Without urgent action, all of Earth's inhabitants face an existential threat. Health professions education should therefore prepare learners to not only practice in a changing world, but authentic educational activities should also develop competencies for global and planetary citizenship.

Health professionals work at the “coalface” of the impacts of a changing climate, dealing, for example, with heat stress from abnormally high temperatures, smoke inhalation from wildfires, and malnutrition and starvation as a result of droughts and floods. At the November 2021 Congress of the Parties (COP26), for the first time, health was placed at the center of the climate conversation, with global health professionals offering 10 recommendations for a healthy future (5). But, in delivering healthcare, healthcare systems in many countries “do harm” to people and the environment—locally, nationally, and internationally—through upstream requirements and activities (e.g., mining of natural resources for equipment, water usage for drug development, and manufacture) and downstream greenhouse gas (GHG) emissions, waste, and pollution (6, 7). Australia's healthcare system contributes about 7% (global average = 4.4%) of national emissions (8), making it a top four *per capita* emitter (6), driven largely by coal- and gas-based energy and reliance on single-use items (8). The net-zero trajectory for the Australian healthcare sector, largely through mitigation, has thus been identified as “steep” (9). But as trusted professionals (10), all health professions, and by implication, all health professionals, should be environmentally accountable for their personal and professional activities (11). Such citizenship requires, amongst other competencies, systems and design thinking, as well as collective action and leadership. Health professions education thus has a responsibility to graduate individuals who are prepared to take action in a changing world (12) in which existing inequities will be exacerbated in a warming climate (13). They also should be able to educate patients, for example, about the co-benefits (to self, community, and the planet) of exercise (e.g., walking or cycling to work) and sustainable diets (e.g., reducing red meat intake; increasing dietary fruit and vegetables) (14). Healthcare professionals thus have a duty to advocate for larger-scale institutional, political, and economic change to address the health inequity and environmental challenges that have arisen from historic and ongoing systemic injustices (15).

The United Nations (UN) 2030 Sustainable Development Goals (SDGs) comprise a set of 17 interrelated Goals with Targets and Indicators that promote equitable actions for a sustainable future. In higher education, the Sustainable Development Solutions Network's (SDSN) 2017 guide for Australia, New Zealand, and the Pacific region (16), and the more recent, “Accelerating Education for the SDGs in Universities” guide (17), provide frameworks and case studies to support SDG implementation in universities. In the United States, the Association for the Advancement of Sustainability in Higher Education (AASHE) has incorporated the SDGs into its STARS program, developing campus metrics for reporting SDGs in terms of operations and curricula (18), while The Times Higher Education Impact Rankings publish global performance tables which assess universities against the SDGs (19).

The SDG framework can inform health professions education (20). The common generic Indicator 4.7.1 for SDGs 4 (*Quality Education*), 12 (*Sustainable Production and Consumption*), and 13 (*Climate Action*)—*Global citizenship education and education for sustainable development are mainstreamed in national education policies, curricula, teacher education, and student assessment* (20, 21)—can be applied to health professions education. The UN defines global citizenship as “the umbrella term for social, political, environmental, and economic actions of globally minded individuals on a worldwide scale” (20). Implementing the SDGs, including global citizenship, in higher education is, however, not without its challenges, ranging from poor sustainability literacy and a lack of support from management (18). The concept of global citizenship has also been contested (22), and global citizenship education challenged for potentially masking national or local responsibilities (23). However, we live in an interconnected world, and what happens in one country or region can have global impacts. Global carbon inequality is one such example. In calculating this inequality from 1990 to 2019, Chancel (24) reported that in 2019, the top 10% of the world's population emitted 48% of the total carbon emissions, while the bottom 50% contributed only 12% of the emissions. With carbon emissions a significant contributor to our changing climate, the brunt of which is borne by the poorest countries such as Afghanistan and Bangladesh (25), global citizenship is a necessary educational outcome (26).

It is worth noting, however, that the SDGs are now 7 years old and with the dire state of many of the planet's ecosystems, some of those who advocated for global citizenship as a competency are now calling for a paradigm shift, e.g., from “global nursing” to “planetary nursing” (26–28). Calls for planetary health integration in medical education (29) are being translated into action, such as a recent *Frontiers in Public Health* publication describing a student-driven planetary health curriculum (30). Global citizenship (social focus) can now be supplemented with planetary citizenship (environmental focus). For Turner, “identifying as a planetary citizen means seeking to understand humanity's environmental footprint and trying to do something about it. The aggregate effect of planetary citizenship across multiple levels of organization (individual, civil society, national, global) will lead to purposeful change at the planetary scale” (31).

This submission describes the outcomes of a second-year learner-centered planetary health assignment in a five-year Doctor of Medicine (MD) program at Bond University, Gold Coast, Australia, after 5 years of implementation (2018–2022) (32). Underpinned by SDG13 (*Climate Action*), self-selected teams of 4–6 students submit a protocol (with feedback) for a proposed “product” (creative output designed to deliver a message to a particular audience), which they then develop and submit. Five pairs of academic graders select their best two products (*n* = 10), voting for the Academics' Choice Award. The teams are then invited to pitch their product (5 min) to their peers, who choose the People's Choice winner and runner-up.

## Pedagogical framework, principles, and competencies

The planetary health assignment is underpinned by four primary considerations: *Global citizenship* (a Bond University graduate attribute and an Indicator for several SDGs), the *SDGs* (including ecological justice), *teamwork*, and *learner-centeredness* (33).

### Global citizenship

**Figure 1:**
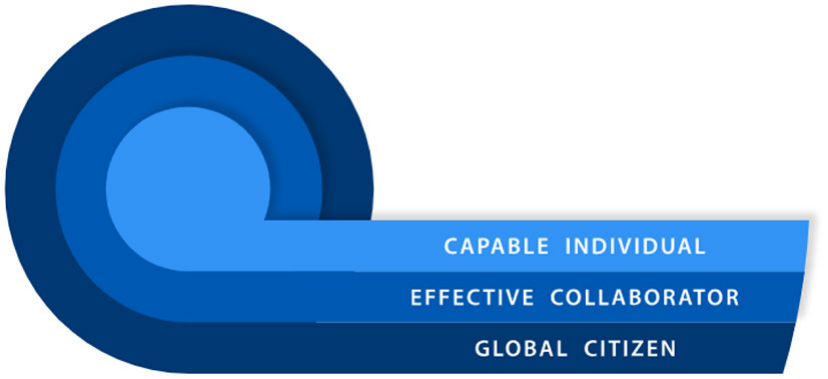
Bond University graduate attributes: capable individual, effective collaborator, and global citizen (34).

Bond University has three graduate attributes (Figure 1): Graduates need to be *capable individuals, effective collaborators*, and *global citizens* (34). As global citizens, graduates should thus take responsibility for their actions and understand the need for personal accountability. They should also employ integrity, professionalism, and ethical decision-making in all aspects of their enterprise. Viewed through a planetary health lens, graduates should therefore be accountable for their environmental footprints, personally and professionally, as individuals and as health professionals, recognizing the impacts locally, nationally, and globally. Considering the deteriorating state of the global (35) and the Australian natural environment (36), and considering the above-average environmental footprint of the Australian healthcare sector (6), environmental accountability in the health professions is paramount. In addition, with Australia being the second dirtiest *per capita* economy in the world (37), all Australians have a personal responsibility to mitigate their footprint.

### The SDGs, specifically SDG13 (*Climate Action*), including ecological justice

In 2019, Bond University became a signatory to the SDGs. Both prior to this and subsequently in response to the agreement, academics are integrating these Global Goals into various educational programs across the University. Considering the range of current environmental threats to human health (e.g., heat, smoke inhalation, deteriorating ecosystems, etc.), ensuring that all health professional students are prepared to practice (and take action) is an educational priority. For this planetary health assignment, SDG13 (*Climate Action*) is the focus, supported by at least one other SDG of choice.

Early in Year 1, Bond medical students are introduced to how “planetary health” has been integrated across the five-year Medical Program. Over the past few years, planetary health integration in medical education has been student-driven [e.g., Planetary Health Report Card (38, 39)], with a recent example of such a planetary health curriculum being that of Warren Alpert Medical School (30). While planetary health can be viewed through several lenses, with Bond University located in Australia, where First Nations Peoples were the traditional custodians of all of Australia's ecosystems for about 65,000 years before colonization by European settlers began, we have applied an Indigenous lens to planetary health. Students are provided with this Redvers (40) description: “Planetary health as a “field” is primarily a Western construct as Indigenous Traditional Knowledge systems have no clear separation of self or that of the community and the ecosystem at large. This means that the meaning and application of planetary health are directly rooted in community values based on protocols for living in harmony with all that have existed for thousands of years.”

With rich Traditional Knowledges and an Aboriginal and Torres Strait Islander *Social and Emotional Well-being Framework* (41), and connection to “Country” an integral part of this Framework, ecological justice is embedded not only in this planetary health assignment but also as a scheduled session in the same semester. Ecological justice holds that non-human beings such as animals and plants also have entitlements such as an adequate habitat, which is in line with Indigenous views of First or Natural Laws (42).

The SDGs recognize that a range of factors, from colonization, unequal development, and unjust wealth inequalities have not only marginalized many but have also come at the expense of our natural environment. Under the slogan of “Leave No-one Behind,” the SDGs aim to eradicate poverty and hunger, reduce inequality within and among states, and provide a “plan of action for people, planet, and prosperity” (33) by prioritizing Indigenous knowledge systems and advancing education for women sustainably. Ecological sustainability and environmental protection are not achievable without addressing people's basic needs and ensuring a more equitable (which has not been the case to date) sharing of the planet's limited resources so that people can fulfill their potential in a healthy environment.

“Leaving no-one behind” and eliminating poverty depends, however, on protecting land rights, eliminating gender and racial inequalities, and recognizing the interdependence of human well-being and healthy, optimally biodiverse ecosystems. The ecological justice movement thus extends environmental justice by ascribing rights to land, species, and ecosystems (43). The right of an ecosystem to be protected against ecocide is of particular significance. Recent examples of ecological justice in action include the legal recognition of the personhood of the Whanganui River in New Zealand and Mutuhekau Shipu (Magpie River) in Canada (44).

### Teamwork

Teamwork is fundamental for safe and high-quality healthcare (45), and learning to work as a member of a team is a key skill in health professions education. While health professionals may not always be able to choose their team members, for this learner-centered planetary health assignment, students were free to do so. For most small group work in the Program, they are assigned to teams.

### Learner-centeredness

Learner-centeredness is a key consideration of this planetary health assignment (46, 47). With the only stipulation of the assignment that of using SDG13 (*Climate Action*) Targets and Indicators to address pressing issues, students had choices in terms of:

- Forming teams (4–6 students), electing a team leader, and agreeing on a team name,
- Choosing at least one additional SDG (with Targets and Indicators),
- Choosing an “issue” (problem identification),
- Selecting an appropriate audience, and,
- Deciding on the message delivery format, i.e., a product (submitted for grading).

## Learning environment

Planetary health is integrated across the five-year curriculum in the Bond University MD Program. It begins in the second week of the first year when students are introduced to the key principles (e.g., the SDGs, definitions, planetary boundaries, etc.) of planetary health (*Introduction to Planetary Health*) that frame the longitudinal curriculum integration in terms of environmental sustainability, reconnecting with and protecting and restoring our natural environment. The same week, students are guided through the rationale for adopting an *Eco-biopsychosocial Model of Health and Well-being*, framed by a strong and nested sustainability model (48). Year 2 builds on these foundations and includes the planetary health assignment currently described as well as an ecological justice session. In Year 3, elements of sustainable healthcare are integrated into patient scenarios, e.g., environmental footprint of anesthetic gases, multidose inhalers, and radiological imaging. Environmentally sustainable healthcare spirals into the Year 5 General Practice rotation, with a session during which students explore their experiences of sustainability (or lack thereof) in clinical practice. In addition, some students choose to complete a 12-week Planetary Health subject offered in a Master of Healthcare Innovation for their MD Project that runs across Years 4 and 5.

Below, we provide further details relating to the design and measured outcomes of this planetary health assignment which is assigned 10% of the year grade.

### Assignment learning aims and outcomes

The main aims of the assignment include teams applying at least two SDGs (SDG13, plus at least one other SDG of choice) to a planetary health “issue” they identify and use systems and/or design thinking to develop a product for a specific audience to address the identified problem. A self-study Storyline 360 (Articulate Global, New York, USA) module available on the learning management system outlines design and systems thinking and provides examples of how these “thinkings” have been applied to healthcare. With choice being a focus of this learner-centered assignment, several second-year Learning Outcomes can be applied, primarily in three of the four Medical Program domains: Health and Society (HS), Science and Scholarship (SS), and Professionalism and Leadership (PL) as listed below:

- **HS04:** Outline the range and scope of health promotion and disease prevention programs and evaluate them using knowledge of human behavior.
- **HS09:** Explain common population health screening and prevention approaches, including the use of technology.
- **SS16:** Examine evidence-based approaches to diagnosis, prognosis, and risk.
- **PL02:** Apply the principles of autonomy, beneficence, non-maleficence, and justice to patient care and problem-solving.
- **PL05:** Foster a duty of care while promoting social justice and resource stewardship.
- **PL13:** Demonstrate behaviors in accordance with codes and policies that define legal, ethical, and professional responsibilities.
- **PL14:** Demonstrate the competencies to think critically and logically, using initiative and the best available evidence when facing challenges, solving problems, and making decisions.
- **PL15:** Explain how lifestyle and professional activities may have a negative impact on others locally and globally.

### Pedagogical format: Three graded components

Initially (2018–2020), the planetary health assignment contributed 3% of the year grade. In line with a transition to more programmatic assessment (49), the assignment now (2021–2022) contributes 10% of the year grade. There are currently three graded components: A proposal (4%), a product (5%), and a reflective critique (1%). Proposals are submitted at the end of Week 3 in the middle 12-week semester in a three-semester academic year. Products and reflective critiques are submitted at the end of Week 8. Selected teams pitch their products in Week 11.

### Proposal, product, and reflective critique grading

Five pairs of markers comprising Medical Program academics, supported by Higher Degree Research students who are tutors in the Program, are responsible for grading the proposals, products, and reflective critiques. Each pair is responsible for overseeing 7–8 teams. In terms of grading proposals, each member of the academic pair independently grades the proposals using the assigned rubric based on Boyer's expanded scholarship criteria (50) before meeting to collate and upload their written feedback to individual teams via the learning management system. The grading rubric assesses proposals in terms of providing clear goals for the product, including a well-researched and referenced literature review, appropriately applying systems and/or design thinking, and feasibility of the proposed product in the time allocated, the articulated benefit and impact of the proposed product, and the quality of the written submission. Teams can discuss this feedback with their graders should clarification be required. No marks are assigned at this stage. The same grading process is followed for the products and the reflective critiques. The products are assessed on whether, based on feedback received, the team has created a quality product (e.g., error-free, engaging, properly referenced, etc.) that is appropriate for the intended audience. The quality of their reflection (reflective critique) is also assessed. In Week 10, academics meet to discuss the grading of all three components (inter-rater reliability). Each pair nominates two teams (based on product quality and appropriateness) to make a 5-min pitch to their peers at a conference in Week 11. Academics vote for the Academics' Choice Award (first place and runner-up). Following the team pitches, the cohort votes for the People's Choice Award. Certificates are awarded to the four winning teams. The Medical Program then donates AUS$50 to the four teams' chosen environmental organizations.

### Self-reported personal and professional development

Immediately following the People's Choice Award voting, students anonymously respond to seven statements which evaluate whether intended and advertised outcomes have been met. They report on their awareness of the impact of a changing environment, their responsibility (values, attitudes) in mitigating and advocating in this regard and they also indicate whether the assignment has contributed to the development of a range of skills.

This team-based, learner-centered planetary health assignment is innovative in that it encourages learners to think globally in terms of the impact of their personal and future professional actions, and, using the SDGs, engages them in proposing solutions to climate change. This submission thus aims to answer the following question: *Did this planetary health assignment, designed to engage teams of learners to “take action” on a pressing global issue (climate change), meet the intended outcomes?*

### Ethics

Ethics for the study was approved by the Bond University Human Research Ethics Committee (CM03517).

## Results

The data archived over the 2018–2022 period (*n* = 162 teams) were analyzed to identify trends in terms of the planetary health “issues,” the second SDG selected to target alongside SDG13 (*Climate Action*), the intended audience, and the final product format.

### Main “issues” identified

**Figure 2:**
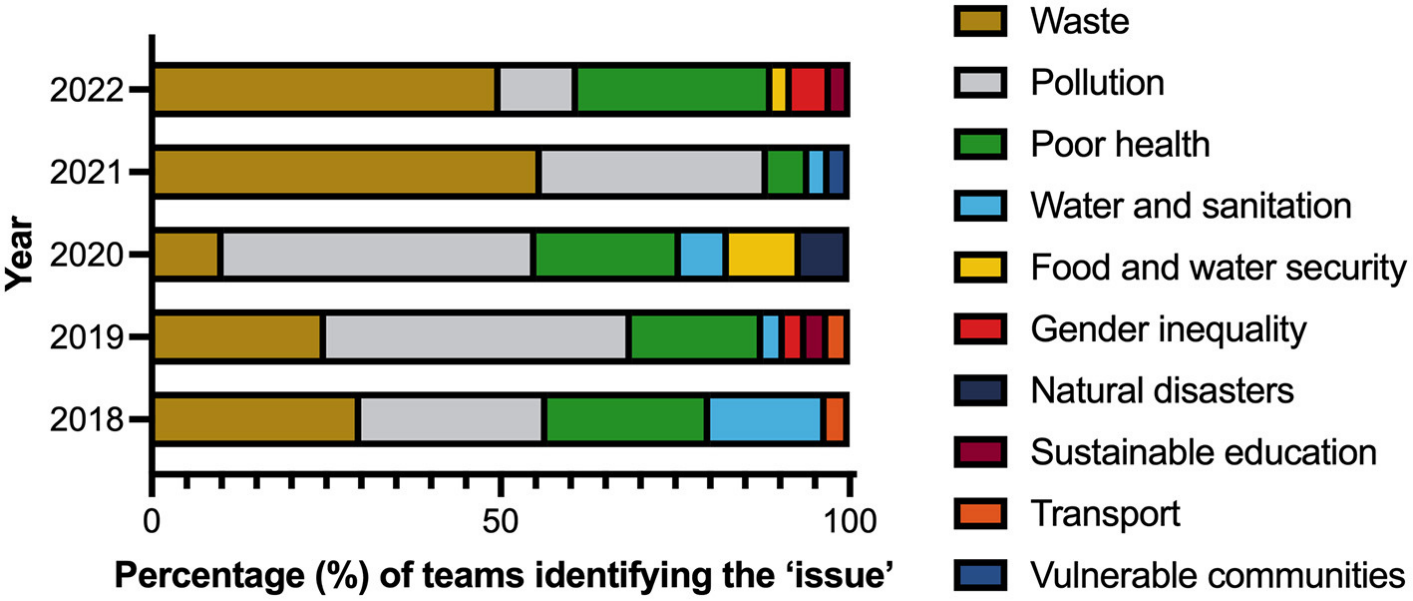
Percentage of teams identifying a range of “issues” across a five-year period (2018–2022, *n* teams = 162: 2018 = 30, 2019 = 32, 2020 = 30, 2021 = 34, 2022 = 36).

Figure 2 depicts the planetary health “issues” identified over the five-year period (2018–2022). Collectively across the 5 years (*n* = 162 teams), waste (35.4% of all teams), pollution (31.1%), and poor health (19.3%) were the most frequently identified “issues.” The subcategories addressed under the issue of waste (*n* = 57 teams) included healthcare (82.5%), food (14%), and general household waste (3.5%). On a year-by-year basis, waste was the most common “issue” identified in 2018 (30% of 30 teams), 2021 (55.9% of 34 teams), and 2022 (50% of 36 teams) while pollution was the most frequently identified issue in 2019 (43.8% of 32 teams) and 2020 (44.8% of 30 teams). Types of pollution (*n* = 50 teams) included air (32%), land (28%), water (26%), and GHG emissions (14%). Poor health (*n* = 32 teams) was the second most common issue identified in 2020 (20.7%) and 2022 (27.8%), which addressed topics such as poor lifestyle (e.g., diet and exercise for 40.6% of these teams), communicable disease (25.0%), mental health (21.9%), anti-microbial resistance (6.3%), sexual health (3.1%), and hand hygiene (3.1%).

### Second SDG selected

**Figure 3:**
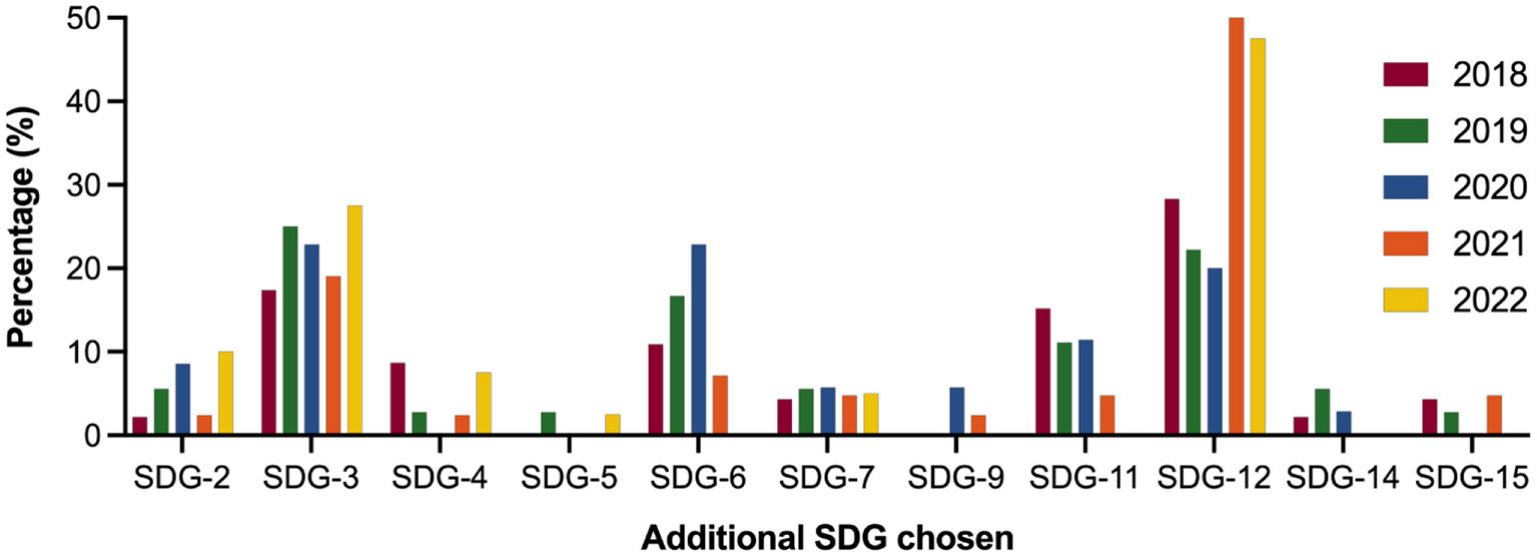
Second SDG (% of teams in each cohort) selected for the five-year period (2018–2022, *n* teams = 162: 2018 = 30, 2019 = 32, 2020 = 30, 2021 = 34, 2022 = 36). As all teams had to use SDG13, data for this goal is not included.

As described earlier, the primary aim of the assignment was for teams to use SDG13 (*Climate Action*) to advocate for action on climate change. Over the 5 years, the most selected additional SDGs were:

- SDG12 (*Sustainable Production and Consumption*) −41% of teams, mostly relating to healthcare emissions and waste
- SDG3 (*Health and Well-being*) −22%, involving the impact of air pollution
- SDG6 (*Clean Water and Sanitation*) −15% (Figure 3).

There was reasonable consistency across the 5 years, although SDG12 selection increased in the last 2 years, accounting for 62% (2021) and 53% (2022) of team choices. There were no significant differences in terms of the additional SDG selected across the cohorts (Kruskal-Wallis test with Dunn's multiple comparison assessment).

In terms of the least selected SDGs, no teams selected SDG1 (*No Poverty*) and SDG10 (*Reduced Inequalities*) over the 5 years. SDG8 (*Decent Work and Economic Growth*) was selected by one team in 2021 and SDG16 (*Peace, Justice, Strong Institutions*) by one team in 2018. Two teams selected SDG17 (*Partnership for the Goals*) in 2018. It is worth noting that a few teams did choose a third SDG to fully address the intended scope of their “product.” These have not been included in the analysis. An example of this would be SDG5 (*Gender Equality*), in addition to SDG12 in relation to environmentally friendly feminine hygiene products.

### Intended audience and product format

The “issues” identified influenced who the intended audience was and hence the format of the product. Table 1 provides the composite data for the intended audiences and the product delivery format for the 5 years. The most targeted audiences were local, state, or federal governments (24.7% of teams), healthcare professionals (i.e., doctors, nurses, pharmacists) (24.1%), and the public (21.6%). In 2018, just over one-third of teams chose governments as their target audience, while in 2021, 41% of teams chose healthcare professionals as their target audience.

**Table 1:** Intended audience and product format for the five-year period (2018–2022).

	**Frequency per year (%)**					
**Audience**	**Overall**	**2018**	**2019**	**2020**	**2021**	**2022**
	**(*n* = 162)**	**(*n* = 30)**	**(*n* = 32)**	**(*n* = 30)**	**(*n* = 34)**	**(*n* = 36)**
Government	24.7	36.7	25.0	26.7	8.8	27.8
Healthcare professionals	24.1	23.3	15.6	16.7	41.2	22.2
Public	21.6	23.3	21.9	23.3	26.5	13.9
University	19.1	6.7	21.9	26.7	14.7	25
Schools	3.1	10	3.1	0.0	0.0	2.8
Pharmaceutical companies	2.5	0.0	0.0	0.0	2.9	8.3
Vulnerable populations	2.5	0.0	3.1	0.0	2.9	0.0
Non-profit organization	1.2	0.0	6.3	0.0	0.0	0.0
Farmers	1.2	0.0	3.1	0.0	2.9	0.0
**Product format**						
Video	26.7	13.3	28.1	20.0	41.2	27.8
Information pamphlet	18.6	16.7	31.3	36.7	8.8	2.8
Written proposal	16.8	36.7	9.4	6.7	8.8	25.0
Slideshow presentation	15.5	26.7	18.8	20.0	11.8	2.8
Website	14.3	3.3	9.4	6.7	17.6	30.6
Mobile app	6.2	3.3	0.0	6.7	8.8	11.1
Podcast	1.9	0.0	3.1	3.3	2.9	0.0

*n* = number of teams with 4–6 students per team.

Across the 5 years, the most frequent product formats were video (26.7%), information pamphlets (18.6%), and written proposals (16.8%). The trend across the 5 years suggests the development of digital skills, with a decrease in slideshow presentations and information pamphlets, and an increase in more sophisticated digital skills such as creating websites, videos, and “apps.” Interestingly, there was a resurgence of written proposals in 2022 (25.0%), following a decline in 2019 and 2021.

### Self-reported personal and professional development

Table 2 summarizes learners' self-reported personal and professional development after completing the assignment. The average response rate for the five-year period was 51.6% (of the total 788 students completing the assignment). Overwhelmingly, students reported personal and professional development in terms of their awareness of the environment as a determinant of health and their responsibility to “take action” on climate change. Their responses also indicated an improvement in a range of skills, such as teamwork (80.8%), information-handling (72.8%), problem-solving (71.2%), and digital technology (62.0%).

**Table 2:** Self-reported awareness of environmental responsibility and skills development by completing the planetary health assignment (2018–2022).

	**Agree**	**Not sure**	**Disagree**	***n***
	**(%)**	**(%)**	**(%)**	**(2018–2022)**
***Values, behavior, and attitudes:*** **This assignment has…**				
Made me more aware of the relationship between the environment and health and well-being	83.1	10.1	6.8	268
Made me think how I can personally reduce my environmental footprint (i.e., be part of the solution by mitigating)	83.0	9.8	7.2	240
Made me aware of my future role as a health professional (i.e., advocacy, education about environmental factors affecting health)	83.8	10.6	5.6	224
***Skills development:*** **This assignment has developed my …**.				
Teamwork skills	80.8	14.1	5.1	230
Information-handling skills	72.8	19.6	7.6	217
Problem-solving skills	71.2	17.3	11.5	207
IT and/or technology skills	62.0	26.3	11.7	247

*n* = individual students.

## Discussion

Although the warning bells about climate change and health rang many years ago, with Costello and colleagues warning in 2009 that climate change was the greatest threat to human health, potentially undoing the last 50 years of progress (51), medical education has generally been slow to include global health issues such as climate change and air pollution in the curriculum. A 2019–2020 International Federation of Medical Students' Associations (IFMSA) survey of over 2,800 medical schools in 112 countries found that few medical curricula had included climate change (15%) or air pollution (11%) (39). In response to these curricular omissions, medical students have been proactive in setting up organizations (e.g., Medical Students for a Sustainable Future, M4SF) (52) and developing curricular frameworks and learning outcomes (53), as well as producing training manuals and issuing policy statements (e.g. IFMSA) (54). In 2019, medical students developed the Planetary Health Report Card, a self-evaluation tool comprising five metrics to guide universities and health professions schools' self-audits to identify areas requiring attention (38).

As concerned academics, we recognized the need to graduate Australian doctors suitably equipped to deal with the consequences of rising temperatures and adverse weather events, who also need to be sensitive to the impacts on marginalized (therefore requiring prioritization) individuals and populations in an already unequal world. It seemed appropriate then in 2018 to use the SDGs (also referred to as the Global Goals for collective action, i.e., global citizenship) to underpin the planetary health assignment. With climate change a pressing global issue not only for people but also for the planet, SDG13 (*Climate Action*) framed the assignment. The authentic assessment was also designed to include a range of activities that would allow students to not only increase their knowledge and develop skills [e.g., problem-solving, systems thinking (55)] but which would also foster the acquisition of values and attitudes (e.g., civil responsibility) to be able to “take climate action” both in their personal lives as global citizens, but also collectively as future health professionals. These students' future professional body, the Australian Medical Association (56), in conjunction with an advocacy group, Doctors for the Environment Australia, recently released a communique, *Governments and the healthcare sector must lead on climate change*, advocating an 80% reduction in carbon emissions by 2030 and a net-zero Australian healthcare system by 2040. This is a major undertaking considering that the Australian healthcare system was listed as a top four *per capita* emitter in 2019 (7). A steep decarbonization trajectory is thus required (9).

### Practical implications: Were the intended outcomes met?

In describing the measured outcomes after 5 years of implementation of an authentic, team-based, learner-centered planetary health assignment completed by second-year students in the Bond University Medical Program, the key question to be answered is: *Did this planetary health assignment, designed to engage teams of learners to “take action” on a pressing global issue (climate change), meet the intended outcomes?* Considering that the students were studying medicine, the outcomes in terms of the “issues” identified, and the SDGs selected over the 5 years of implementation are not surprising: Waste (mostly in healthcare), pollution and poor health led to SDG12 (*Sustainable Production and Consumption*) being the most frequently identified secondary SDG, followed by SDG3 (*Good Health and Well-being*), and SDG6 (*Clean Water and Sanitation*). More sustainable production and reduced consumption would lead to less waste and less pollution (e.g., cleaner air) and hence improved health outcomes. In terms of SDG6, “taking action” generally related to addressing “issues” in communities most likely to be impacted, such as in the Solomon Islands and remote Australia (Indigenous communities), where, as future Year 5 students, their MD capstone healthcare immersion would provide opportunities for advocacy. Further evidence of advocacy involved “taking action” in the form of creating informational videos, websites, and writing proposals directed at individuals or groups in positions of power, i.e., local and national government ministers and healthcare professionals, to take note of and hopefully respond.

Using self-reported data on the impact of the assignment across the 5 years, more than 80% of the students who responded reported a heightened awareness of the relationship between the environment and health and well-being, how they could personally reduce their environmental footprint (i.e., be part of the solution by mitigating), and that their future health professional roles would require advocating for action. Again, these findings align not only with global citizenship as a Bond University graduate attribute (20) but also as an outcome of several SDGs (15, 16), and reflect planetary citizenship.

Students reported improvement in a range of skills relevant to their future clinical practice: Teamwork (80.8%), information-handling (72.8%), problem-solving (71.2%), and information technology (IT) (62.0%). These self-reported improvements align with other Bond University graduate attributes of becoming *capable individuals* and *effective collaborators* who show strong interpersonal skills, can lead or contribute in effective teams, create, think critically, problem solve, and demonstrate information literacy (34). An interesting find was that only 62% of students reported IT (digital technology) skills improvement. There are several likely explanations. One might be that not all teams chose to develop products requiring these skills, e.g., compare writing a policy proposal with developing a website. With technology now embedded in many medical curricula (57, 58), such as the need for these students to develop a website the previous semester, many already might consider themselves to be “digitally literate.” In addition, the COVID-19 pandemic accelerated this literacy as remote learning during lockdowns required the adaptation of lecture delivery, assignments, and assessment (59). There are reported benefits from remote delivery formats (60), although this often requires upskilling of both faculty and students (57).

This assignment is unique in the Bond Medical Program (and probably in many medical curricula) in several respects:

- The learner-centered design allows students to make several choices, including team members. Mostly during their medical studies, for convenience, individuals are assigned to groups.
- It is authentic in that learners tackle a real-world pressing global issue (climate change) using a global framework (UN SDGs, with Targets and Indicators) for “taking action.” Using the SDGs exposes students not only to the concept of environmental sustainability, but also provides an opportunity to reflect on global inequity from a position of “privilege” as students living and studying in a wealthy country with a large *per capita* footprint individually and in their future profession.
- It is skills-based, from problem-solving to communication, and includes systems and design (61) thinking, both of which have been identified as key skills in healthcare (62). All other skills built into the assignment are important in healthcare.
- Teams receive feedback on their first submission (their proposals), which they can apply immediately in the development of their second submission (their products), leading to high quality outputs. Generally, in assignments, feedback is not provided until after submission.
- Teams create fit-for-purpose products which can be sent to the intended audience, e.g., a policy document to a minister of health or a website available to the public.

### Lessons learned

From our perspective as educators, this assignment, although labor-intensive, is an extremely hopeful exercise, and proof-positive that we can educate and support students to engage in harm reduction by addressing systemic inequity and taking care of the planet. While some students may already be advocates and ecological change agents, most will discover these concepts through the longitudinal integration of planetary health in their curriculum. Many will, however, require institutional support and a proper grounding in the dynamics of climate change, ecological justice, and the socio-economic determinants driving the current crisis. When provided, such as with this Year 2 assignment, our findings suggest that a number of teams have advocated for change on pressing issues by addressing their concerns and suggestions to politicians or other individuals who have the power to change the *status quo*. As health professions educators, our findings also offer considerable reason for hope. In addition to improving the understanding of the determinants of health for themselves and their future patients, we believe that our students have also gained an understanding of how healthcare currently “does harm” by contributing to both upstream (production; manufacture) and downstream (waste; pollution) damage to ecosystems and to communities who are culturally and spiritually connected to land or Country. We believe that many learners have developed an increased understanding of the need to treat the natural world, other species, and other cultures as morally considerable. In our judgment, most now appreciate the compelling need to integrate planetary health and ecological justice principles into their future work as health professionals.

Based on our findings, we are of the opinion that this planetary health assignment, as part of longitudinal planetary health curriculum integration, supports students to not just be global citizens but also to be planetary citizens, developing the knowledge, skills, values, and attitudes to tackle the Code Red crisis that all of Earth's inhabitants are facing (2–4). Our approach not only aligns with but extends the Bond University graduate outcomes (20) and the SDG education Targets (15, 16).

### Future directions

At this point, our conclusions are based on self-reported development of values and attitudes involving environmental responsibility, advocacy, and skill development. It would be valuable to ascertain whether this assignment (as part of longitudinal planetary health integration) leads to real changes in environmental attitudes and behaviors (rather than perceptions of change) over a longer period.

While the 2030 SDGs embody global citizenship, in the 7 years since the SDGs were created, we believe that citizenship should be extended to the planetary level, involving ecological justice (31). In the Bond University Medical Program, “planetary health” is integrated using an Indigenous lens, which embodies environmental stewardship in line with Natural or First Laws and Traditional Knowledges (42, 63). This is particularly important considering the poor state of Australia's ecosystems. First Nations Peoples ways of knowing, being, and doing are thus vital for sustaining and restoring Australia's declining ecosystems (36).

## Data availability statement

The raw data supporting the conclusions of this article will be made available by the authors, without undue reservation.

## Ethics statement

The studies involving human participants were reviewed and approved by Ethics for the study was approved by the Bond University Human Research Ethics Committee (CM03517). Written informed consent for participation was not required for this study in accordance with the national legislation and the institutional requirements.

## Author contributions

MM: conceptualization, methodology, and project administration. MM, CP, VV, JS, NM, DB, RM, BC, and CM: validation, investigation, and reviewing and editing the publication. MM, CP, JS, NM, and CM: formal analysis. MM, CP, NM, and CM: data curation. MM, CP, NM, RM, BC, and CM: writing the publication. MM, CP, JS, and CM: visualization and figures. MM and CM: supervision. All authors approved the final manuscript for publication.

## Conflict of interest

The authors declare that the research was conducted in the absence of any commercial or financial relationships that could be construed as a potential conflict of interest.

## Publisher's note

All claims expressed in this article are solely those of the authors and do not necessarily represent those of their affiliated organizations, or those of the publisher, the editors and the reviewers. Any product that may be evaluated in this article, or claim that may be made by its manufacturer, is not guaranteed or endorsed by the publisher.
